# RETRACTION: NBPF4 Mitigates Progression in Colorectal Cancer Through the Regulation of EZH2‐Associated ETFA

**DOI:** 10.1111/jcmm.70716

**Published:** 2025-07-24

## Abstract

**RETRACTION**: ChenW. , DiZ. , ChenZ. , NanK. , GuJ. , GeF. , LiuJ. , ZhangH. , and MiaoC. , “NBPF4 Mitigates Progression in Colorectal Cancer Through the Regulation of EZH2‐Associated ETFA,” Journal of Cellular and Molecular Medicine 25, no. 18 (2021): 9038–9050, 10.1111/jcmm.16867.34405537 PMC8435418

The above article, published online on 18 August 2021 in Wiley Online Library (wileyonlinelibrary.com), has been retracted by agreement between the authors; the journal Editor‐in‐Chief, Stefan Constantinescu; The Foundation for Cellular and Molecular Medicine; and John Wiley and Sons Ltd. The retraction has been agreed following an investigation into concerns raised by a third party, which revealed inappropriate duplications of image panels (Figure 2G) between this and another article published previously by different group of authors in an unrelated scientific context. The authors acknowledged the error, stating that a placeholder image sourced from published literature was mistakenly left in the submitted manuscript. While they cooperated with the investigation and provided partial raw data, this did not sufficiently address the concerns raised. Further review by the journal uncovered additional issues, including inconsistencies in the reported results on EZH2 silencing. Therefore, the editors consider the integrity of the data and the validity of the study's conclusions compromised and decided to retract the article. The authors apologize for the oversight and any inconvenience caused.

**RETRACTION**: W. Chen , Z. Di , Z. Chen , K. Nan , J. Gu , F. Ge , J. Liu , H. Zhang , and C. Miao , “NBPF4 Mitigates Progression in Colorectal Cancer Through the Regulation of EZH2‐Associated ETFA,” Journal of Cellular and Molecular Medicine 25, no. 18 (2021): 9038–9050, 10.1111/jcmm.16867.34405537 PMC8435418

The above article, published online on 18 August 2021 in Wiley Online Library (wileyonlinelibrary.com), has been retracted by agreement between the authors; the journal Editor‐in‐Chief, Stefan Constantinescu; The Foundation for Cellular and Molecular Medicine; and John Wiley and Sons Ltd. The retraction has been agreed following an investigation into concerns raised by a third party, which revealed inappropriate duplications of image panels (Figure 2G) between this and another article published previously by different group of authors in an unrelated scientific context. The authors acknowledged the error, stating that a placeholder image sourced from published literature was mistakenly left in the submitted manuscript. While they cooperated with the investigation and provided partial raw data, this did not sufficiently address the concerns raised. Further review by the journal uncovered additional issues, including inconsistencies in the reported results on EZH2 silencing. Therefore, the editors consider the integrity of the data and the validity of the study's conclusions compromised and decided to retract the article. The authors apologize for the oversight and any inconvenience caused.

